# Multiacquisition with variable resonance image combination T2 (MAVRIC SL T2) for postoperative cervical spine with artificial disc replacement

**DOI:** 10.1038/s41598-022-23358-8

**Published:** 2022-11-09

**Authors:** Ro Woon Lee, Yeo Ju Kim, Daehyun Yoon, Seunghun Lee, Jeongah Ryu

**Affiliations:** 1 Department of Radiology, Inha University Hospital, 27 Inhang-Ro, Jung-Gu, Incheon, 22332 South Korea; 2 Department of Radiology, Hanyang University Seoul Hospital, College of Medicine, Hanyang University, 222-1, Wangsimni-Ro Seongdong-Gu, Seoul, 04763 South Korea; 3 Department of Radiology, Stanford University, Stanford, CA 94305 USA; 4 Department of Radiology, Hanyang University Guri Hospital, College of Medicine, Hanyang University, Guri, 11923 South Korea

**Keywords:** Health care, Medical research

## Abstract

Anterior cervical discectomy with artificial disc replacement (ADR) is an effective treatment of cervical degenerative disc disease. However, postoperative MRI due to recurrent neck/radicular pain is limited due to severe metallic artifacts of artificial disc instrument. Multiacquisition with variable resonance image combination selective T2 (MAVRIC SL T2) has been developed as an MRI technique for metal artifact reduction but has not been evaluated for the postoperative cervical spine with ADR. In our study, we compared MAVRIC SL T2 with the fast spin echo (FSE) T2-weighted sequence (T2WI), which was an essential MR sequence for evaluation of the cervical neural structure, for metallic artifact reduction in the post-operative cervical spine with ADR. Our study revealed MAVRIC SL T2 showed smaller signal void areas, less distortion and signal pile-up, and was more clinically useful than T2WI (*p* < 0.05). The spinal cord, vertebral bodies, both neural foramina, and anterior paravertebral soft tissue were significantly more visible with MAVRIC SL T2 than with T2WI (*p* < 0.05). MAVRIC SL T2 might be a useful technique for the evaluation of postoperative cervical spine with ADR and complements T2WI in the evaluation of the spinal cord and nerve roots which were important structures for post-operative recurrent neck/radicular pain.

## Introduction

Anterior cervical discectomy with artificial disc replacement (ADR) is an effective treatment of cervical degenerative disc disease with less loss of segment motion and less risk of adjacent level degeneration than anterior cervical discectomy and fusion (ACDF)^1^. However, when it comes to post-operative MR imaging, the ADR has a great challenge in post-operative radiological diagnosis, comparing with ACDF^2^. The artificial disc instrument which used in ADR distorts the magnetic field severely resulting in severe metallic artifacts and making it difficult to assess bony and neurologic structures at the implanted and adjacent levels, regardless of the metal property^2,3^. Multiacquisition with variable resonance image combination selective (MAVRIC SL) have been developed for metallic artifact reduction. Our previous study demonstrated short tau inversion recovery (STIR) using MAVRIC SL technique is significantly decreased metallic artifact of the artificial disc instrument comparing with STIR without MAVRIC SL^4^. Therefore, we wondered whether fast spin echo T2 weighted sequence combined with MAVRIC SL (MAVRIC SL T2) can also replace fast spin echo T2 weighted sequence with superior metallic artifact reduction for the postoperative cervical spine with ADR^4^. The purpose of our study was to evaluate MAVRIC SL T2 for metallic artifact reduction in MRI in the postoperative assessment of the cervical spine with ADR compared with the fast spin echo (FSE) T2-weighted sequence (T2WI).

## Results

### Quantitative analysis

The areas of signal voids of MAVRIC SL T2 in both the axial and sagittal planes were significantly smaller than those of T2WI (*p* = 0.018 for axial plane, 0.012 for sagittal plane, Table 1).

**Table 1:** The areas of signal voids of MAVRIC SL T2 in both the axial and sagittal planes.

	Axial			Sagittal		
	T2WI	MAVRIC SL T2	*p* value	T2WI	MAVRIC SL T2	*p* value
Average (mm^2^)	4982.9	1531.1	0.012	4149.5	2252.6	0.018
Standard deviation	2016.16	429.8		1128.7	721.2	

### Qualitative analysis

#### Interobserver agreement of independent analysis of three readers

The k values of interobserver agreement for the qualitative analysis are shown Table 2. Most variable showed moderate to almost perfect agreement between at least two readers. Axial images have a relatively low k value of clinical usefulness.

**Table 2:** The *k* values of interobserver agreement for the qualitative analysis.

Variables	*k*-value		
	R1 vs. R2	R2 vs. R3	R1 vs. R3
**Distortion**			
Axial	0.75	0.88	0.86
Sagittal	0.61	0.75	0.74
**Signal pile-up**			
Axial	0.81	0.74	0.75
Sagittal	0.63	0.53	0.7
**Visualization of anatomic structure**			
**Axial**			
Anterior subarachnoid space	1	1	1
Spinal cord	0.73	0.73	0.57
Neutral foramina	0.66	0.76	0.66
Anterior paravertebral soft tissue	0.5	0.54	0.69
**Sagittal**			
Anterior subarachnoid space	0.79	0.89	0.89
Spinal cord	0.62	0.83	0.57
Neutral foramina	0.92	0.92	0.92
Anterior paravertebral soft tissue	0.87	0.83	0.78
Upper vertebral body	0.67	054	0.57
Lower vertebral body	0.69	0.81	0.68
**Clinical usefulness**			
**Axial**			
Spinal cord compression in operated level	0.54	0.87	0.65
Nerve root compression in operated level	0.49	0.68	0.55
Spinal cord signal change	0.55	0.6	0.7
**Sagittal**			
Spinal cord compression in operated level	0.71	0.79	0.83
Nerve root compression in operated level	0.87	0.95	0.82
Disc herniation or bulging in the adjacent disc level	0.79	0.77	0.78
Spinal cord signal change	0.8	0.86	0.86

*R1* reader 1, *R2* reader 2, *R3* reader 3.

#### Comparison between T2WI and MAVRIC SL T2 by the scores from consensus reading

**Figure 1:**
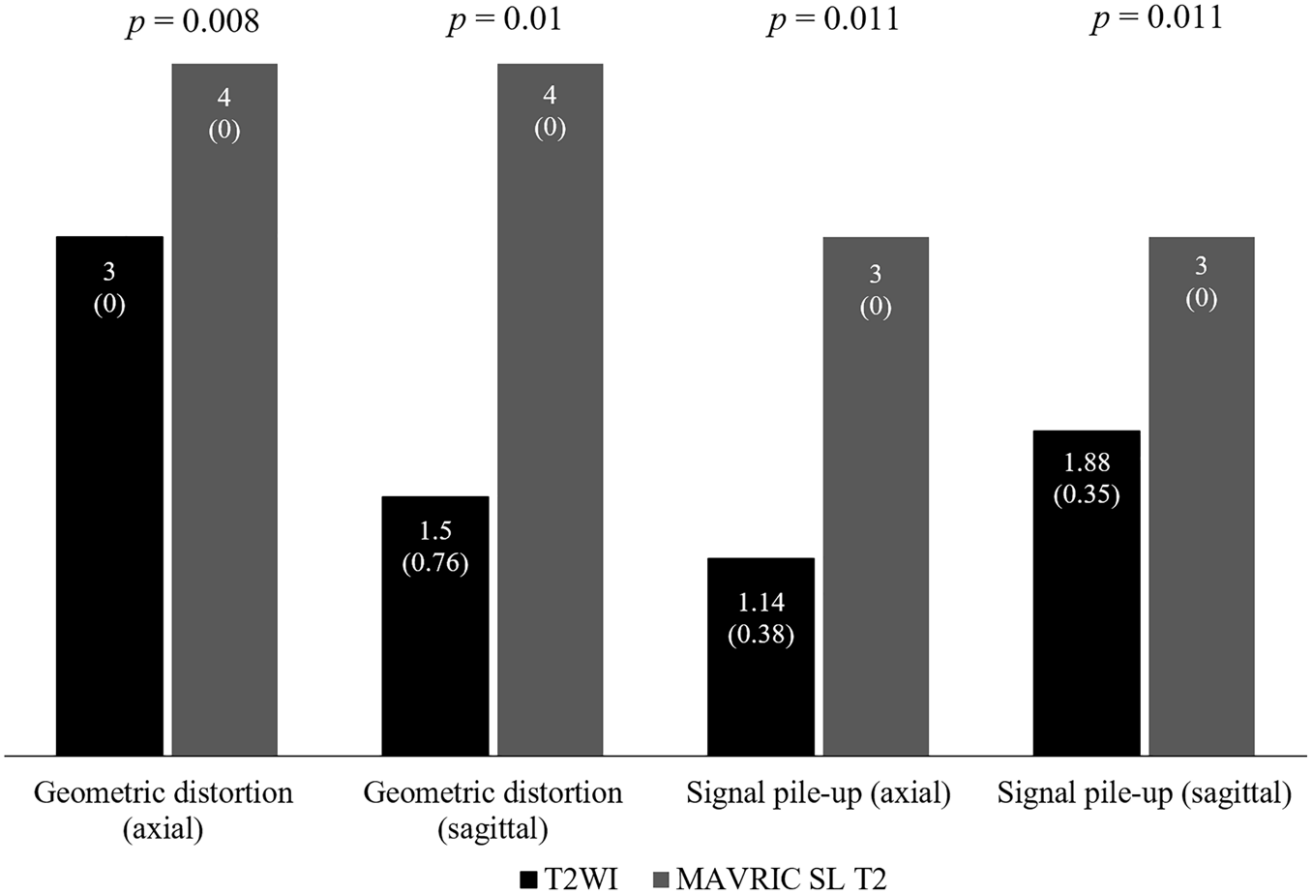
Average scores of the consensus reading for geometric distortion and signal pile-up of the axial and sagittal plane of T2-weighted sequence and multiacquisition with variable resonance image combination selective T2-weighted sequence (MAVRIC SL T2). Numbers on the top of the bar are the average score and the numbers in parentheses for each bar are the standard deviation. The *p* value for each category is noted at the top of the graph.

**Figure 2:**
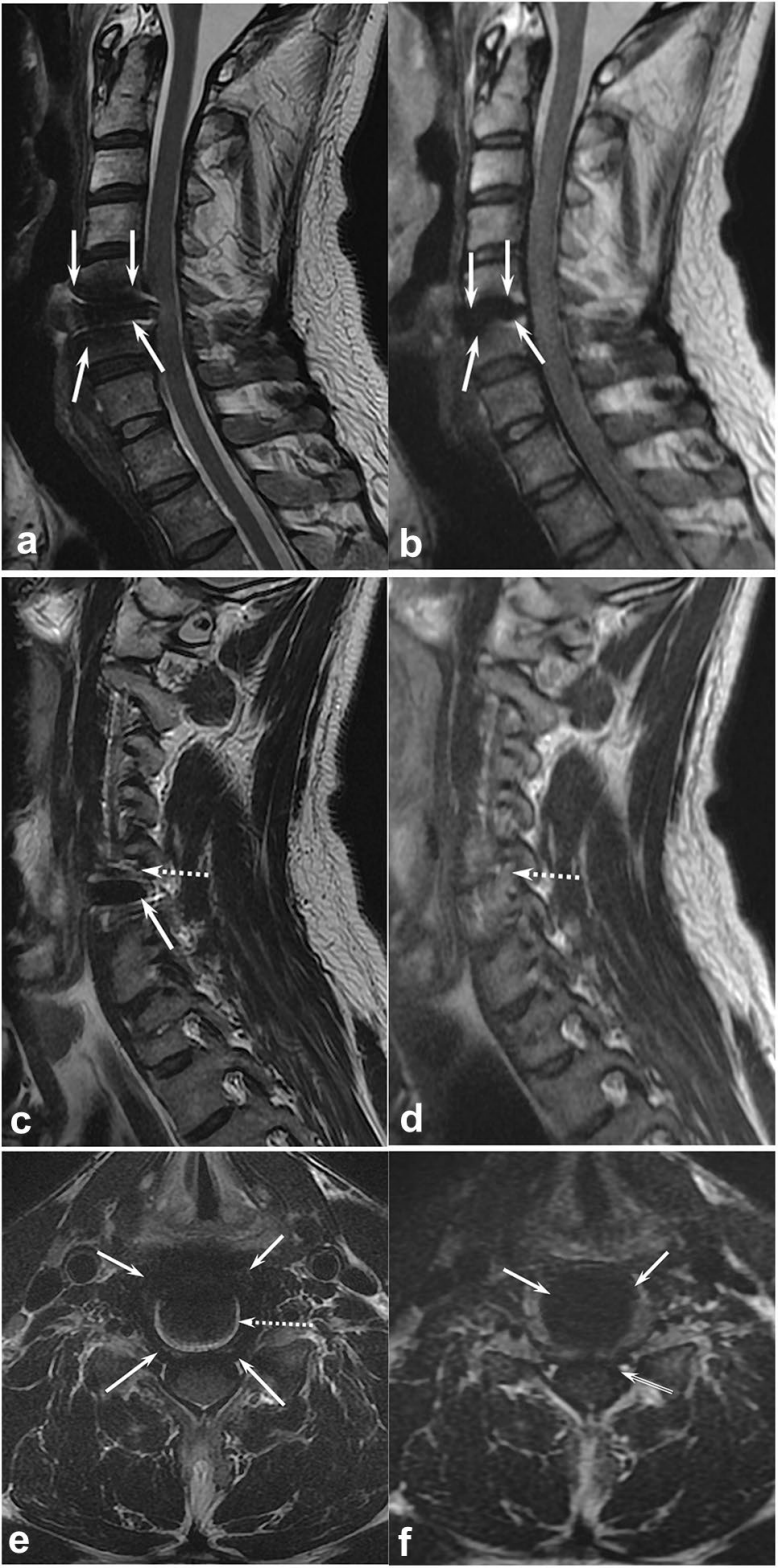
Comparisons of T2-weighted sequence (T2WI) (**a**, **c**, **e**) and multiacquisition with variable resonance image combination selective T2-weighted sequence (MAVRIC SL T2) (**b**, **d**, **f**) of the cervical spine of a volunteer (V3). (**a**) Mid-sagittal T2WI shows a metallic artifact (arrows) that obliterated anterior paravertebral soft tissue, upper and lower vertebral bodies, and anterior surface of the spinal cord. (**b**) Mid-sagittal MAVRIC SL T2 showed a markedly decreased signal void area (arrows) with nearly no geometric distortion and minimal signal pile-up. However, severe blurring was observed in the spinal cord. (**c**) Parasagittal T2WI at the left foramen level shows a signal void (arrow) obliterating more than half of the neutral foramen. Note the mild geometric distortion of the nerve root (dashed arrow). (**d**) Parasagittal MAVRIC SL T2 at the left foramen level visualized completely normal neutral foramen with no metallic artifact and no distortion of the nerve root (dashed arrow). (**e**) Axial T2WI at the operated level shows signal voids (arrows) obliterating the anterior subarachnoid space, anterior portion of the spinal cord, anterior paravertebral soft tissue, and both neutral foramina. Note the signal pile-up artifacts (dashed arrow). (**f**) Axial MAVRIC SL T2 showed a marked decrease in signal void (arrows) and almost no signal pile-up artifacts. However, the anterior subarachnoid space is obliterated by the low signal intensity of the cerebrospinal fluid pulsation artifact (double lined arrow). Severe blurring was noted in the spinal cord, with decreased T2 contrast.

MAVRIC SL T2 showed significantly less geometric distortion and significantly fewer signal pile-up than T2WI in both the axial and sagittal planes (*p* < 0.05, Fig. 1). In particular, for the geometric distortion, MAVRIC SL T2 showed almost no distortion (Figs. 1 and 2).

**Figure 3:**
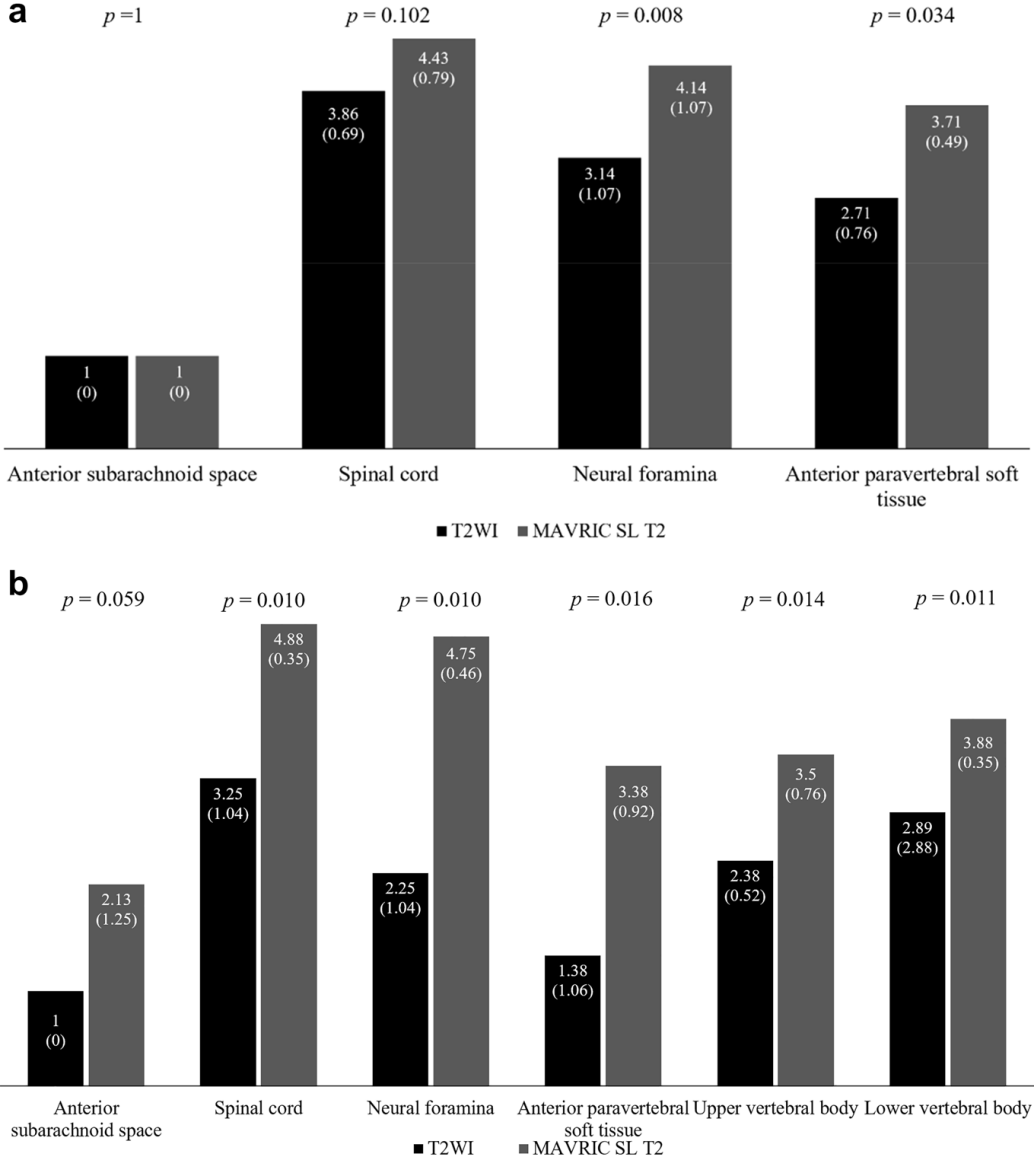
Average scores of the consensus reading for the visualization of anatomic structure of T2-weighted sequence (T2WI) and multiacquisition with variable resonance image combination selective T2-weighted sequence (MAVRIC SL T2) in the axial (**a**) and sagittal planes (**b**). Numbers on the top of the bar are the average score and the numbers in parentheses for each bar are the standard deviation. The *p* value for each category is noted at the top of the graph.

The average scores for anatomic structure visualization in T2WI and MAVRIC SL T2 are shown in Fig. 3. In the axial plane (Fig. 3a), the neural foramina and anterior paravertebral soft tissue were significantly clearer in MAVRIC SL T2 than in T2WI. In the sagittal plane, all but the anterior subarachnoid space was significantly better visualized in MAVRIC SL T2 than in T2WI (*p* < 0.05, Fig. 3b). In particular, the spinal cord and neutral foramina were almost completely visualized in the sagittal plane of the MAVRIC SL T2 (Figs. 2 and 3).

**Figure 4:**
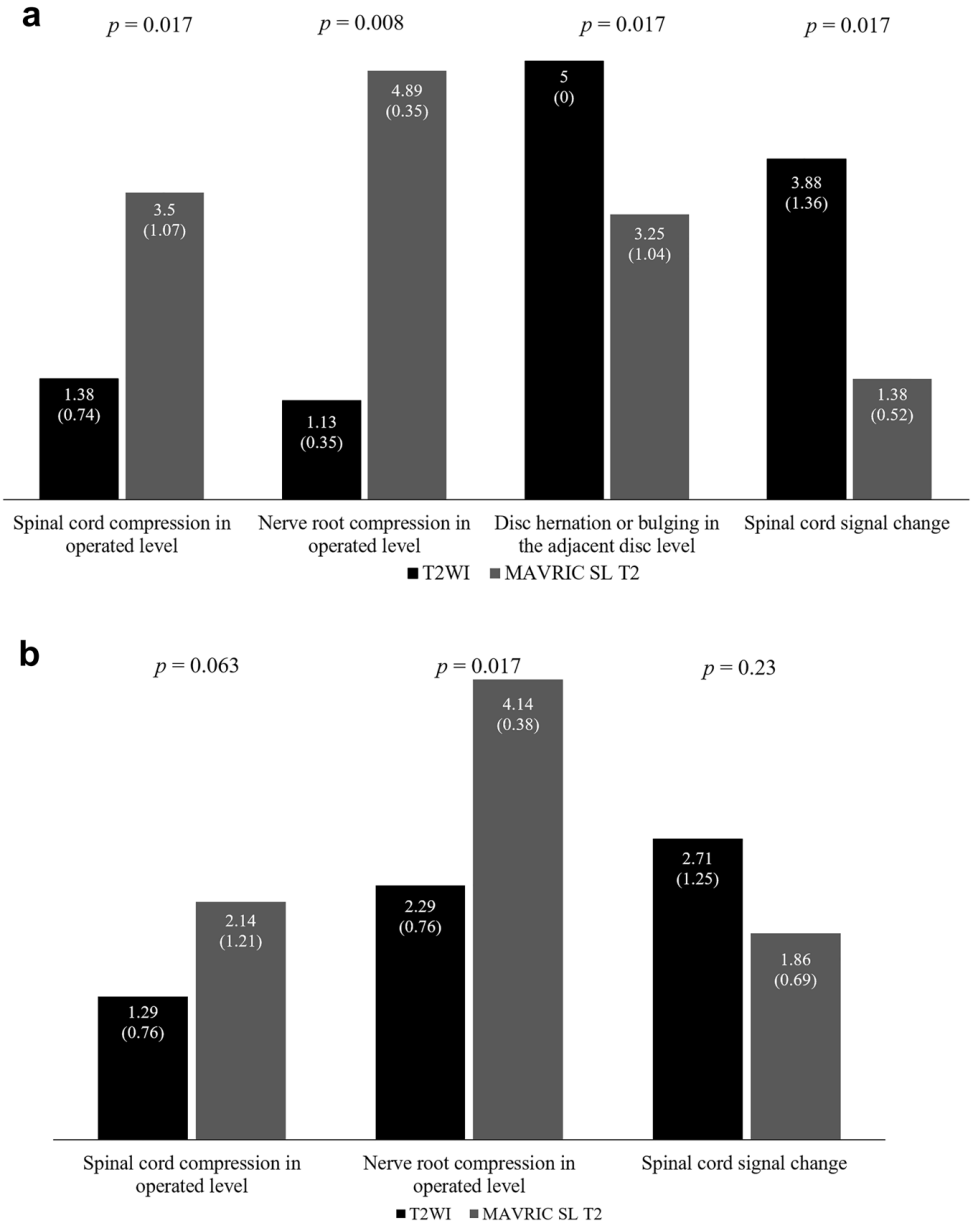
Average scores of the consensus reading for clinical usefulness of T2-weighted sequence (T2WI) and multiacquisition with variable resonance image combination selective T2-weighted sequence (MAVRIC SL T2) in the sagittal (**a**) and axial planes (**b**). Numbers on the top of the bar are the average score and the numbers in parentheses for each bar are the standard deviation. The *p* value for each category is noted at the top of the graph.

In terms of clinical usefulness, MAVRIC SL T2 showed significantly better clinical usefulness for the evaluation of spinal cord and nerve root compression at the operated level than T2WI (*p* < 0.05) in the sagittal plane (Fig. 4a). However, it showed poor performance for spinal cord signal changes. Nevertheless, T2WI was significantly more useful for the evaluation of disc herniation or bulging in the adjacent disc level and spinal cord signal change than MAVRIC SL T2 (*p* < 0.05, Fig. 4). In the axial plane, MAVRIC SL T2 showed better clinical usefulness for nerve root compression only at the operated level (*p* = 0.017, Fig. 4b).

## Discussion

In our study, MAVRIC SL T2 effectively reduced areas of signal void, geometric distortion, and signal pile up, resulting in better visualization of anatomic structures, which is in line with previous studies^4–7^. In particular, sagittal MAVRIC SL T2 clearly visualized the spinal cord and neural foramina which are essential structures for the evaluation of recurrent spondylotic myelopathy and radiculopathy. In addition, the anterior paravertebral soft tissue and upper and lower vertebral bodies, which are commonly involved in postoperative infection, were better visualized in MAVRIC SL T2 but severely obliterated in T2WI. Although postoperative infection was mainly evaluated in fat-suppressed fluid-sensitive sequences and enhanced T1-weighted images, MAVRIC SL T2 may have a complementary role in the visualization of anatomic details.

However, in terms of the clinical usefulness, the spinal cord signal change was insufficiently visualized in MAVRIC SL T2 due to blurring and decreased T2 contrast. MAVRIC SL utilizes a longer ETL of 32 and 3D-FSE acquisition, which can result in increased blurring compared to 2D-FSE (ETL = 16)^6,7^. This could be an important limitation for detailed evaluation of the cervical spine, which has a relatively small anatomic structure compared to extremities^6,7^. The T2 contrast in both MAVRIC-SL T2 and T2WI is forged by exploiting the T2-induced signal decay during data sampling along the echo train. The lower refocusing flip angle of MAVRIC SL T2 compared to T2 WI (75 vs. 142–160) results in the slower signal decay along the echo train because smaller refocusing flip angles allow more transverse magnetization to be restored to the longitudinal axis, where the signal decays at a much slower T1 relaxation time as shown in Busse et al.^8^ Therefore, the overall T2-contrast of MAVRIC SL T2 was not as strong as that of T2WI. This might cause an undesirable impact on the evaluation of spinal cord signal change. In axial plane, the spinal cord compression in operated level was also show poor performance in MAVRIC SL T2. MAVRIC SL T2 is not equipped with gradient-moment nulling, which mitigate the CSF flow artifact in T2WI and gradient echo sequences. Therefore, MAVRIC SL T2 showed strong CSF pulsation artifacts, showing dark signal intensity of the CSF within the anterior subarachnoid space, which limits evaluation of any compressive lesion to the spinal cord, especially in the axial plane. A long scan time is also thought to contribute to strong CSF pulsation artifact. MAVRIC SL obtains images using 3D-FSE acquisition with an increased number of excitations to acquire the data for each excited slabs, resulting in long scan times compared to 2D-FSE techniques^5–7,9^. A long scan time is a significant drawback of MAVRIC SL T2 because patients with severe pain may not be able to complete the MRI scan or have severe motion artifacts.

There are several limitations in our study. First, we did not analyze T1 weighted image (T1WI) which is a routine sequence for evaluation of cervical spine^10,11^. Although T1WI is excellent for evaluating bone marrow abnormalities, it is less sensitive for evaluating of spinal cord and nerve root abnormalities that are important for postoperative neck pain. Therefore, we focused on effectiveness of MAVRIC SL T2 compared to T2WI, which was an essential MR sequence for evaluation of the cervical neural structure in the post-operative cervical spine. Second, the number of volunteers included in this study was too small. Third, the diagnostic performance of post-operative complication or recurrent disc pathology among sequences was not conducted because we did not include patients with symptoms. However, considering the long scan time of MAVRIC SL T2, which may be difficult for patients to bear, our preliminary study may be meaningful in verifying the effect of the metallic artifact reduction of MAVRIC SL prior to a large-scale clinical study. Forth, we analyzed most of the artifacts semi-qualitatively, except for the area of the signal void. Therefore, we performed independent analysis and calculated inter-reader reproducibility for the reliability of the semi-qualitative analysis. Fifth, the inter-reader agreement of axial images in clinical usefulness was relatively lower than that of sagittal images. We assumed that strong CSF pulsation artifacts in axial images might affect image interpretation among readers about spinal cord and nerve root compression. Due to the small number of volunteers, even a slight difference in agreement can significantly change the kappa value, so verification with a large population study is required in the future.

In conclusion, MAVRIC SL T2 might be useful for the reduction of metallic artifacts in the postoperative cervical spine with ADR, especially for the reduction of signal loss and distortion, and may complement T2WI in the evaluation of spinal cord and nerve root compression at the operated level.

## Materials and methods

GE Healthcare (Waukesha, WI) provided research support for the implantation and application of MAVRIC SL. This study was prospectively designed and approved by the institutional review board of Inha University Hospital, in accordance with the Declaration of Helsinki. Informed consent was obtained from all participating volunteers.

### Volunteer enrollment and MRI scan

Eight volunteers (5 males and 3 females; mean age, 50.88 years) who underwent ADR using Prodisc C (DePuy Sythes, Raynham, MA, USA) and without recurrent neck and radicular pain in our hospital would like to participate our study. Written informed consent was obtained for all volunteer in accordance with the institutional review board. Table 3 showed the demographic data of the volunteers. Two volunteers performed anterior interbody fusion before ADR due to cervical spondylotic myelopathy (Table 3). All volunteers underwent MRI scans with axial and sagittal T2WI and MAVRIC SL T2 sequences. A 3 T MR system (Architect, General Electric, Waukesha, WI, USA) was used with a head and neck coil. Scans covered the whole cervical bony structure in the sagittal plane and the operated level in the axial plane. A calibration scan was performed for MAVRIC SL T2 to determine the number of spectral bins, which varied per volunteer (between 16 and 24 in our study). The imaging parameters for the calibration scan were as follows: repetition time (TR)/echo time (TE), 2433.2/10; receiver bandwidth (RBW), ± 125 kHz; field of view (FOV), 40 cm; acquisition matrix, 128 × 32; section thickness (ST), 6 mm; echo train length (ETL), 16; and scan time, approximately 1 min and 57 s. Paralled imaging (data-driven parallel imaging reconstruction known as ARC, or Autocalibrating Reconstruction for Cartesian imaging) was only applied on MAVRIC SL. The detailed MRI protocol is presented in Table 4.

**Table 3:** Demographic data of volunteers (V).

	V1	V2	V3	V4	V5	V6	V7	V8
Sex	Male	Male	Female	Male	Male	Female	Female	Male
Age (years)	54	48	43	60	59	57	40	46
Preoperative diagnosis	Herniated disc	Herniated disc	Herniated disc	Herniated disc	Herniated disc	Herniated disc	Herniated disc	Herniated disc
Operated level	C5-6	C5-6	C5-6	C5-6	C6-7	C5-6	C5-6	C5-6
Previous operation	Anterior interbody fusion at C6-7	None	None	Anterior interbody fusion at C6-7	None	None	None	None
Postoperative period(years)	5	6	3	5	4	5	3	2

**Table 4:** MRI protocol of the volunteer study.

	Axial		Sagittal	
	T2WI (2D)	MAVRIC SL T2 (3D)	T2WI (2D)	MAVRIC SL T2 (3D)
TR (ms)	2945.0	3963	2945.0	3746
TE (ms)	102	80	102	80
FA (°)	142	75	160	75
ETL	16	32	16	32
NEX	4	1	4	1
BW (kHz)	35.75	125	50	125
Matrix size	352 × 256	320 × 192	352 × 256	320 × 192
ST/gap(mm)	3/0	3/0	3/0	3/0
FOV (mm)	140	180	240	240
PIF (phase × slice)	NA	2 × 2	NA	2 × 2
Scan time (min:s)	2:48	5:28	3:14	6:30

*2D* two dimensional, *3D* three dimensional, *TR* repetition time, *TE* echo time, *FA* flip angle, *ETL* echo train length, *NEX* number of excitations, *BW* bandwidth, *ST* section thickness, *FOV* field of view, *PIF* paralled imaging factor, *TI* inversion time, *T2WI* fast spin echo T2-weighted sequence, *MAVRIC SL T2* multiacquisition with variable resonance image combination selective T2-weighted sequence, *NA* not applicable.

### Image analysis

After the MRI scan, the axial and sagittal image datasets from each sequence were re-labeled to remove the original sequence name and randomize. Among the eight volunteers, the axial images of T2 and MAVRIC SL T2 of one volunteer (V5) were excluded from image analysis due to image quality.

#### Quantitative analysis

A musculoskeletal radiologist measured the area of signal loss around the prosthesis in all axial and sagittal sequences using the Maroview PACS system (Maroview 5.4, Infinite, Seoul, South Korea) for quantitative analysis of the area of the signal void. An ROI was drawn around the artifact encompassing the signal void (including the implant) image by image in all axial and sagittal T2WI and MAVRIC SL T2 and the areas of signal voids were summed.

#### Semi-qualitative analysis

Three musculoskeletal radiologists (one had 3 years and the others had 13 years of experience) independently evaluated the image datasets for geometric distortion, signal pile-up, visualization of anatomic structures, and clinical usefulness.

A 4-point scoring system was used for evaluation of geometric distortion and signal pile-up (Table 5)^4^. Distortion was defined as changed or impaired anatomic allocation around the prosthesis^4^. Signal pile-up were defined as a peripheral rim of high signal intensity around the prosthesis^4^.

**Table 5:** Scoring system according to each category of the qualitative analysis of the volunteer study.

Distortion	1: Severe distortion made the anatomic allocation of the implant and clinical diagnosis impossible 2: Distortion moderately impaired anatomic allocation near the metal implant and may confound the clinical diagnosis 3: Distortion mildly altered anatomic contour and does not significantly interfere with clinical diagnosis 4: No distortion present
Signal pile-up	1: Severe signal pile-up with obliteration of normal anatomy and it made clinical diagnosis impossible 2: Moderate signal pile-up with partial obliteration of normal anatomic structure and it may confound the clinical diagnosis 3: Mild signal pile-up without significant effect on normal anatomic structure or signal and it does not significantly interfere with clinical diagnosis 4: No signal pile-up
Visualization of the anatomic structure	1: Less than 25% of anatomic structure is visible 2: 25% to 50% of anatomic structure is visible 3: 50% to 75% of anatomic structure is visible 4: More than 75% of anatomic structure is visible 5: Clear image without any artifacts
Clinical usefulness	1: The sequence is useless 2: The sequence gives minimal information and additional sequence must be needed for diagnosis 3: The sequence gives moderate information and may need additional images for diagnosis 4: The sequence gives major information for diagnosis but have limited image quality 5: The sequence gives sufficient information for diagnosis with good image quality

A 5-point scoring system was used for the assessment of visualization of anatomic structure for the following structures: the anterior subarachnoid space, spinal cord, both neural foramina and anterior paravertebral soft tissue for the axial and sagittal planes; and apposing upper and lower vertebral bodies at the operated site in the sagittal plane (Table 5)^4^.

Clinical usefulness was scored by the amount of information each sequence provided in determining common clinical questions of postoperative neck pain (Table 5). The clinical question was whether this sequence can provide enough information for the presence or absence of spinal cord and nerve root compression at the operated level, disc herniation or bulging at the adjacent level, and spinal cord signal change. All clinical questions were assessed in both the axial and sagittal planes, except for disc herniation or bulging at the adjacent level, which was evaluated in the sagittal plane.

Three weeks after the independent analysis, these three radiologists did the 2nd review of all image sets and re-scored for all qualitative variables with consensus for statistical analysis.

### Statistical analysis

The sum of the areas of the signal void for each sequence in the axial and sagittal planes was tested using the Wilcoxon log-rank test. To evaluate the reliability of qualitative analysis of geometric distortion, signal pile-up, visualization of anatomic structure, and clinical usefulness among three readers, weighted Cohen’s kappa statistic (*k*-value) was calculated to determine the interobserver agreement. The strength of agreement quantified by a kappa statistic was graded as follows: < 0, poor; 0.01–0.20, slight; 0.21–0.40, fair; 0.41–0.60, moderate; 0.61–0.80, substantial; and 0.81–1, almost perfect^12,13^. For comparison between the sequences, the average scores from the consensus reading of the qualitative analysis were compared using the Wilcoxon log-rank test.

All statistical analyses were performed using statistical software (MedCalc, version 10.4.0.0, MedCalc, Ostend, Belgium; SPSS 20, IBM, Armonk, NY). Statistical significance was established at *p* < 0.05 for all tests except weighted Cohen’s kappa statistic.

## Data Availability

The datasets used and/or analysed during the current study available from the corresponding author on reasonable request.
